# Comparison of Cyclic and Linear Poly(lactide)s Using Small-Angle Neutron Scattering

**DOI:** 10.1021/acs.macromol.2c02020

**Published:** 2022-12-13

**Authors:** Philip B. Yang, Matthew G. Davidson, Karen J. Edler, Niamh Leaman, Elly K. Bathke, Strachan N. McCormick, Olga Matsarskaia, Steven Brown

**Affiliations:** ^¶^Institute for Sustainability and ^†^Department of Chemistry, University of Bath, Claverton Down, BathBA2 7AY, United Kingdom; ‡ Centre for Analysis and Synthesis, Department of Chemistry, Lund University, SE-221 00Lund, Sweden; § Institut Laue Langevin, 71 Av. Des Martyrs, 38000Grenoble, France; ∥ Scott Bader, Wollaston, WellingboroughNN29 7RJ, United Kingdom

## Abstract

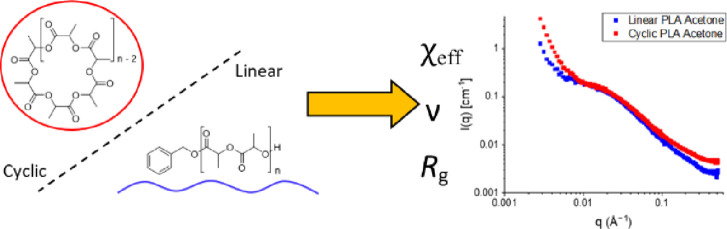

Small-angle neutron scattering (SANS) experiments were conducted on cyclic and linear polymers of racemic and l-lactides (PLA) with the goal of comparing chain configurations, scaling, and effective polymer–solvent interactions of the two topologies in acetone-*d*_6_ and THF-*d*_8_. There are limited reports of SANS results on cyclic polymers due to the lack of substantial development in the field until recently. Now that pure, well-defined cyclic polymers are accessible, unanswered questions about their rheology and physical conformations can be better investigated. Previously reported SANS experiments have used cyclic and linear polystyrene samples; therefore, our work allowed for direct comparison using a contrasting (structurally and sterically) polymer. We compared SANS results of cyclic and linear PLA samples with various microstructures and molecular weights at two different temperatures, allowing for comparison with a wide range of variables. The results followed the trends of previous experiments, but much greater differences in the effective polymer–solvent interaction parameters between cyclic and linear forms of PLA were observed, implying that the small form factor and hydrogen bonding in PLA allowed for much more compact conformations in the cyclic form only. Also, the polymer microstructure was found to influence polymer–solvent interaction parameters substantially. These results illustrate how the difference in polymer–solvent interactions between cyclic and linear polymers can vary greatly depending on the polymer in question and the potential of neutron scattering as a tool for identification and characterization of the cyclic topology.

## Introduction

Cyclic polymers have been mentioned in the literature for decades, but their development has been stunted by synthetic issues and the presence of linear contaminants.^1−4^ However, in the past 15 years, there have been many advances in synthetic methods, which have allowed pure (i.e., no linear contaminants detected in GPC/MALDI-TOF) cyclic polymers to be synthesized with relative ease.^5−10^ Cyclic polymers often exhibit lower viscosities, higher transition temperatures, better thermal stability, and faster crystallization rates compared to conventional linear polymers due to a lack of end groups. The cyclic topology has also been shown to be advantageous in certain catalysis, thermoset adhesive, and drug delivery applications.^1,11^ We have taken interest in using cyclic polymers to improve the commercial potential of bio-based and biodegradable polymers through accessing a wider range of properties.

**Figure 1:**
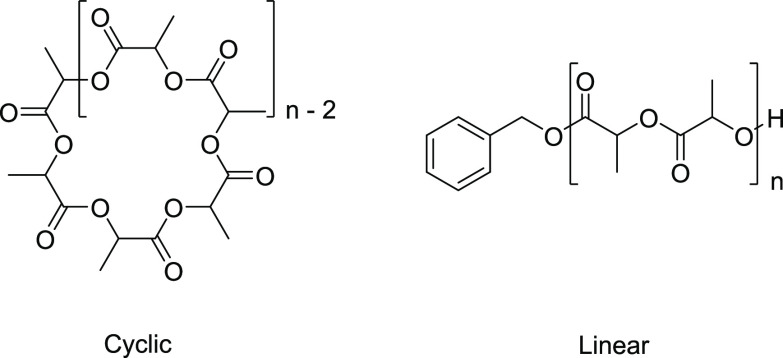
Cyclic and linear topologies of PLA.

The resurgent interest in cyclic polymers means that certain questions have yet to be fully answered, such as their rheological behavior. Specifically, the conformations that cyclic polymers adopt and how they relax stress without free end groups (see Figure 1) are not fully understood. This has been investigated via rheological studies and simulations, but there are varying conclusions, and results have been drastically influenced by linear contaminants.^12−17^

Understanding the physical intricacies of these polymers will allow for better optimization and commercialization of the cyclic topology. For instance, the lack of reptation in cyclic polymers could be useful in applications such as rheology modification.^18^

Polymer–solvent interactions and other physical behaviors can be analyzed using small-angle scattering, either using X-rays (SAXS) or neutrons (SANS). It is known from experiment that the effective polymer–solvent interaction parameter (χ_eff_ or the Flory–Huggins parameter) is lower for a cyclic polymer compared to a corresponding linear polymer.^18^ A lower χ_eff_ value means that the cyclic polymer effectively experiences a more favorable solvent environment. This difference can be rationalized by the smaller radius of gyration (*R*_g_) possessed by the cyclic topology, resulting in a greater local monomer density and more favorable effects from inter/intramolecular interactions. The enthalpic penalty for solvating cyclic polymers is lower as a result. Previous studies have shown that cyclic polymers have lower θ temperatures and higher dissolution limits compared to linear polymers, which supports the idea of a better solvent environment.^19,20^ The extent of the difference in χ_eff_ between linear and cyclic polymers is often dependent on solvent choice, as would be expected.

It is also known that ν, the Flory exponent, which describes how the radius of gyration (i.e., chain size, see eq 1) scales with the molecular weight/degree of polymerization, is slightly larger for the cyclic polymer in a near-θ solvent (ν_cyclic_ = 0.52/0.53 and ν_linear_ = 0.50), but little difference has been reported in a good solvent (near 0.58).^18,21,22^
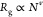
1

Previously reported SANS studies have experimented with cyclic and linear poly(styrene)s in cyclohexane-*d*_12_ at varying temperatures and solvent qualities, showing notable differences when these variables are altered. For instance, the study by Gartner et al. showed that ν for linear polymers decreased monotonically as solvent quality decreases, but this observation was only seen for cyclic polymers in good solvent regimes, while a plateau was observed at near-θ conditions.^18^ However, very few cyclic and linear polymers have been compared using SANS to our knowledge. Known examples include polystyrene and poly(dimethylsiloxane) (PDMS), but they may not represent every polymer case.^18,23,24^ Previous results have also been limited in terms of molecular weights or *q* ranges, and further study is required to fully understand how the cyclic topology behaves in SANS experiments.

Our group has synthesized cyclic polymers of racemic and l-lactides using a modified ring expansion polymerization (REP) catalyzed by a tin(II) catecholate generated in situ from tin octoate, resulting in cyclic polymers with no detectable linear contaminants.^5,25^

**Figure 2:**
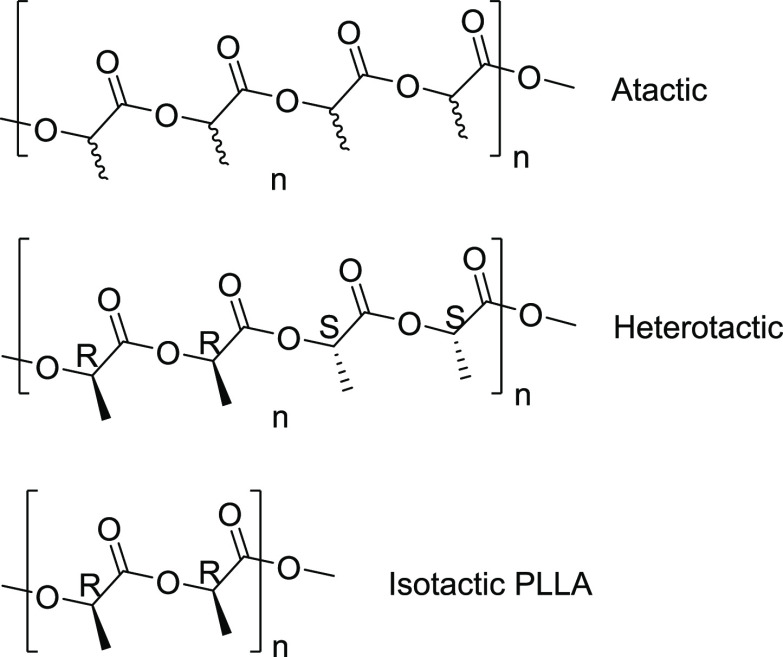
Microstructures of PLA compared in this study with labeled stereocenters.

**Figure 3:**
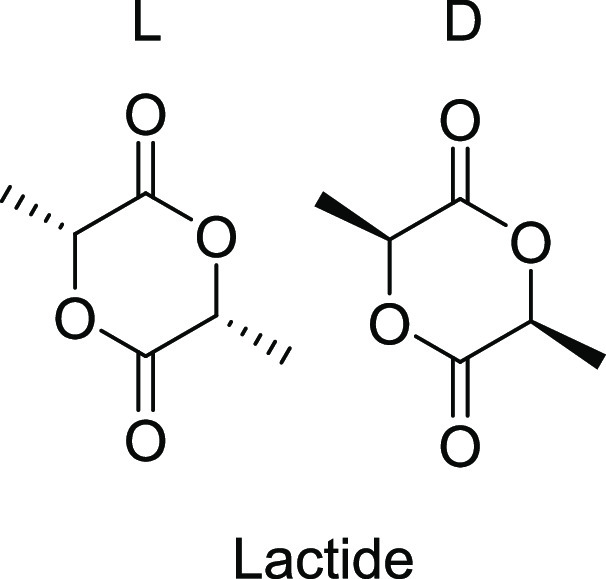
Lactide monomer/s used to synthesize PLA analyzed in this study. The racemic lactide is a 50:50 mixture of l- and d-isomers. Isotactic PLLA was made using l-lactide only.^25^

PLA is one of the most studied cyclic polymers partly because the linear form is the most produced bio-based and biodegradable polymer.^26^ PLA has found use in biomedical and packaging applications and receives constant academic attention because the chirality of lactide allows for variation of polymer microstructures (see Figure 2), which greatly affects physical properties and the degree of crystallinity.^27,28^ Importantly, PLA differs from polystyrene structurally and sterically, to the point where reported molecular weights of PLA from GPC are occasionally corrected to account for this.^29^ Our polymerizations of racemic lactide and l-lactide (see Figure 3) have allowed for experimentation with microstructures, and we have synthesized heterotactically enriched PLA (*P*_r_ = 0.77). Most reported cyclic polymerizations of lactide are of the l-stereoisomer, as PLLA is the commercial form.^6,26,30−32^

Such variation in monomer choice and microstructures (and the effects of changing these variables) is not present in polystyrene. In addition, PLA is a more polar polymer with less steric bulk, which may influence the way in which its rings interact and pack together, which would affect χ_eff_ and ν.

As a result (and due to often reported weak scattering for PLA samples in SAXS),^33^ we report the results of SANS experiments on cyclic and linear poly(lactide) samples at a range of molecular weights, microstructures, and temperatures. This work was done with the aim to compare χ_eff_, ν, and general scattering behaviors to those of previous polystyrene studies while introducing new variables. The use of REP for the synthesis also allows us to see if polymers made using this simple, low-cost approach will give reasonable scattering results, which agree with the expected trends. Successful results would be a good indicator of the synthetic progress made in the synthesis of cyclic polymers in recent years. Alternative ring closure approaches have been found to have a systematic effect on chain conformations and polymer–solvent χ_eff_, but the trends between cyclic and linear topologies were largely unaffected.^18^

## Materials and Methods

### Synthetic Procedures

Atactic cyclic PLA was synthesized using a tin(II) catecholate system generated in situ by the reaction of tin octoate and a catechol. Comparable linear polymers (of *rac*- and l-lactide) were made by replacing the coinitiator with benzyl alcohol, a common coinitiator for linear polymerizations of lactide.^34^ This method was used for the majority of samples used in this study. For cyclic heterotactic PLA and PLLA samples, catalysts and protocols that will be reported elsewhere were used (manuscript in preparation). Cyclic and linear topologies were determined by comparing viscosities in Mark–Houwink plots, using MALDI-TOF and ^1^H NMR to confirm a lack/presence of end groups on the polymers and by comparing glass transition temperatures (*T*_g_’s). Cyclic polymers are known to have noticeably lower viscosities and higher transition temperatures compared to linear counterparts of similar molecular weights, and the extent of these differences is well-known for PLA in particular.^5,6^ Such evidence for cyclic topology can be found in the Supporting Information. Basic characterization of samples used in this study, including molecular weights, probability of racemic enchainment (*P*_r_), and *T*_g_’s, can be found in Table 1.

**Table 1:** GPC Characterization of Poly(lactide) (PLA) Samples Measured in This Work

sample	tacticity	*M*_n_(g mol^–1^)^a^	*M*_w_(g mol^–1^)	*Đ*	*T*_g_ (°C)	*P*_r_
cycle C1	atactic	18,330	29,170	1.59	43.3	0.6
cycle C2	atactic	60,060	88,080	1.47	48.1	0.6
cycle C3	isotactic-PLLA	74,440	135,710	1.82	55.4	N/A^b^
cycle C4	heterotactic	29,130	51,580	1.77	46.9	0.77
linear L1	atactic	19,900	33,480	1.68	42.3	0.6
linear L2	atactic	34,500	54,530	1.58	44.2	0.6
linear L3	isotactic-PLLA	38,040	45,872	1.21	47.7	N/A^b^

a Molecular weight determined by GPC relative to polystyrene standards.

b *P*_r_ unavailable as these samples are polymers of l-lactide only (i.e., no racemic enchainment possible).

#### Cyclic Poly(*rac*-lactide)

3-Methyl catechol (0.0012 g, 0.01 mmol) was added to a dry Schlenk flask under argon before Sn(Oct)_2_ (0.0041 g, 0.01 mmol) was added as a 0.01 M solution in chlorobenzene. *rac*-Lactide (1.4413 g, 10 mmol) was then added under a flow of argon before the closed vessel was immersed in an oil bath preheated to 160 °C for 60 min. The reaction was then stopped, and the colorless, viscous polymer was dissolved in dichloromethane before being precipitated using methanol. The precipitate was recovered and dried in vacuo to remove the residual solvent. [Monomer]:[catalyst] ratios were varied from 100:1 up to 1000:1 for all polymerizations to give polymers of varying molecular weights.

#### Linear Poly(lactide)

Benzyl alcohol (0.0011 g, 0.01 mmol) was added to a dry Schlenk flask under argon before Sn(Oct)_2_ (0.0041 g, 0.01 mmol) was added as a 0.01 M solution in chlorobenzene. *rac*-Lactide (1.4413 g, 10 mmol) was then added under a flow of argon before the closed vessel was immersed in an oil bath preheated to 160 °C for 60 min. The reaction was then stopped, and the colorless, viscous polymer was dissolved in dichloromethane before being precipitated using methanol. The precipitate was recovered and dried in vacuo to remove the residual solvent.

### SANS Measurements

All samples were dissolved in acetone-*d*_6_ or THF-*d*_8_ at 1 wt %, and scattered neutron intensities were collected. Acetone is the θ solvent for PLA and THF is considered to be a “good” solvent for PLA, commonly used for routine analysis in GPC, MALDI-TOF, etc.^6,7,30^ Samples were filled into round quartz cells (“banjo”-type, Hellma, Muelheim, Germany) with a thickness of 2 mm.

SANS measurements were performed on the D11 SANS instrument at the Institut Laue Langevin (ILL), Grenoble, France with a variable temperature sample block for measurements at 15 and 40 °C.^35^ The sample-to-detector distance was varied between 1.7 and 8 m for all samples to cover a range of *q* from 0.018 to 0.52 Å^–1^. For some samples, an additional sample-to-detector distance was employed to give low *q* measurements down to 0.003 Å ^–1^.

The scattering variable *q* is expressed in terms of the neutron wavelength and scattering angle (θ) as *q* = (4π/λ) sin(θ/2) where the neutron wavelength was λ = 6 Å with a full width at half-maximum (FWHM) wavelength spread of 9%. The data were corrected for transmission, flat field, and detector noise (the latter by a measurement of a boron carbide absorber). The scattering of the solvent was subtracted. The data were calibrated to the absolute scale by attenuated empty beam measurements. Data reduction was performed using Mantid.^36^ Raw data were stored in the .nxs format.^37^ Graphs of the reduced SANS data not shown in this manuscript can be found in the Supporting Information.

### RPA Analysis of SANS Data

As mentioned in the paper by Gartner et al.,^18^ the scattering intensity predicted by the RPA for a polymer–solvent system is expressed as:^38−40^
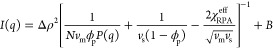
2

Δρ is the difference between the scattering length densities of the polymer and the solvent, *N* is the polymer degree of polymerization, *v*_m_ and *v*_s_ are the volumes of the monomer and solvent particles, respectively, χ_eff_ is the effective Flory–Huggins parameter for the polymer–solvent system, ϕ_p_ is the polymer volume fraction, and *B* is the incoherent background.

The volume fraction ϕ_p_ was fitted to check the validity of the study by comparing it to the known concentration. The results were in good agreement with the expected values, taking into account acceptable error. Whether ϕ_p_ was fitted or not had little effect on the fitting results (see the Supporting Information for an example comparison). The form factor *P*(*q*) for chains with an excluded volume is well-known for linear polymers (see eq 3) and can be expressed in terms of the lower incomplete gamma function, γ, as^38^
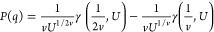
3where *U* = *q*^2^*b*^2^*N*^2ν^/6 and *b* is the statistical segment length, which was a constant value in the fitting of SANS data. However, there is no analytical solution to the form factor for cyclic polymer rings, so it must be evaluated numerically (see eq 4).^40^
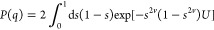
4

The data were fitted using RPA models in SasView (version 5.04) with χ_eff_, ν, and ϕ_p_ as floating variables. Two different RPA models were used for cyclic and linear samples (incorporating either eq 3 or 4 above) to account for the differences in the excluded volume for the two topologies, given that cyclic chains tend to swell more in solution than their linear counterparts and thus have a different form factor. These custom RPA models were kindly provided to the authors by Professor Michael J. A. Hore. Polydispersity was not accounted for in the fitting because molecular weight had only very minor effects on our fitted results, which implies that including dispersity would also have little impact. We also note that the difference in molecular weight across comparable high- and low-molecular-weight samples was substantial enough to result in no overlap in molecular size between these samples. Using identical equations to fit our data also allowed for direct comparison with the previous work on polystyrene.

## Results and Discussion

**Figure 4:**
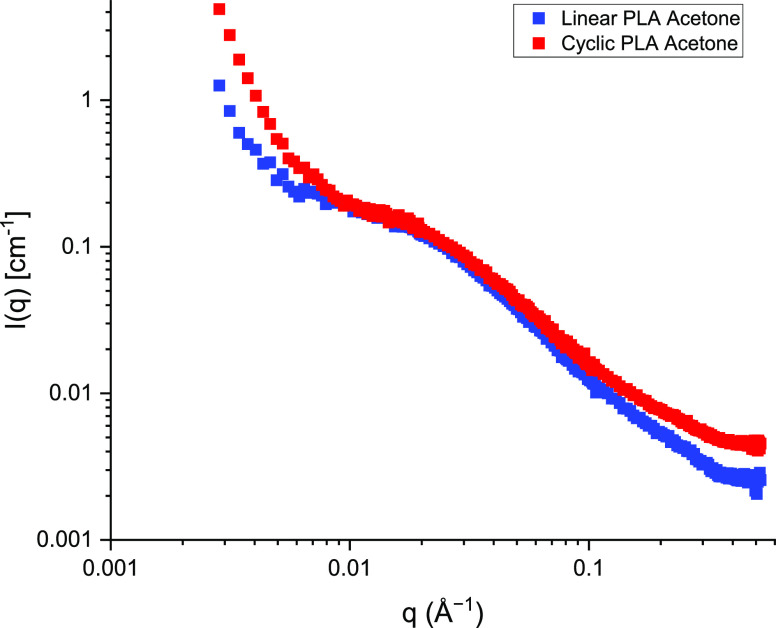
*I*(*q*) vs *q* plot of cyclic and linear poly(lactic acid) (PLA) samples over the full *q* range of 0.003 to 0.52 Å ^–1^.

**Figure 5:**
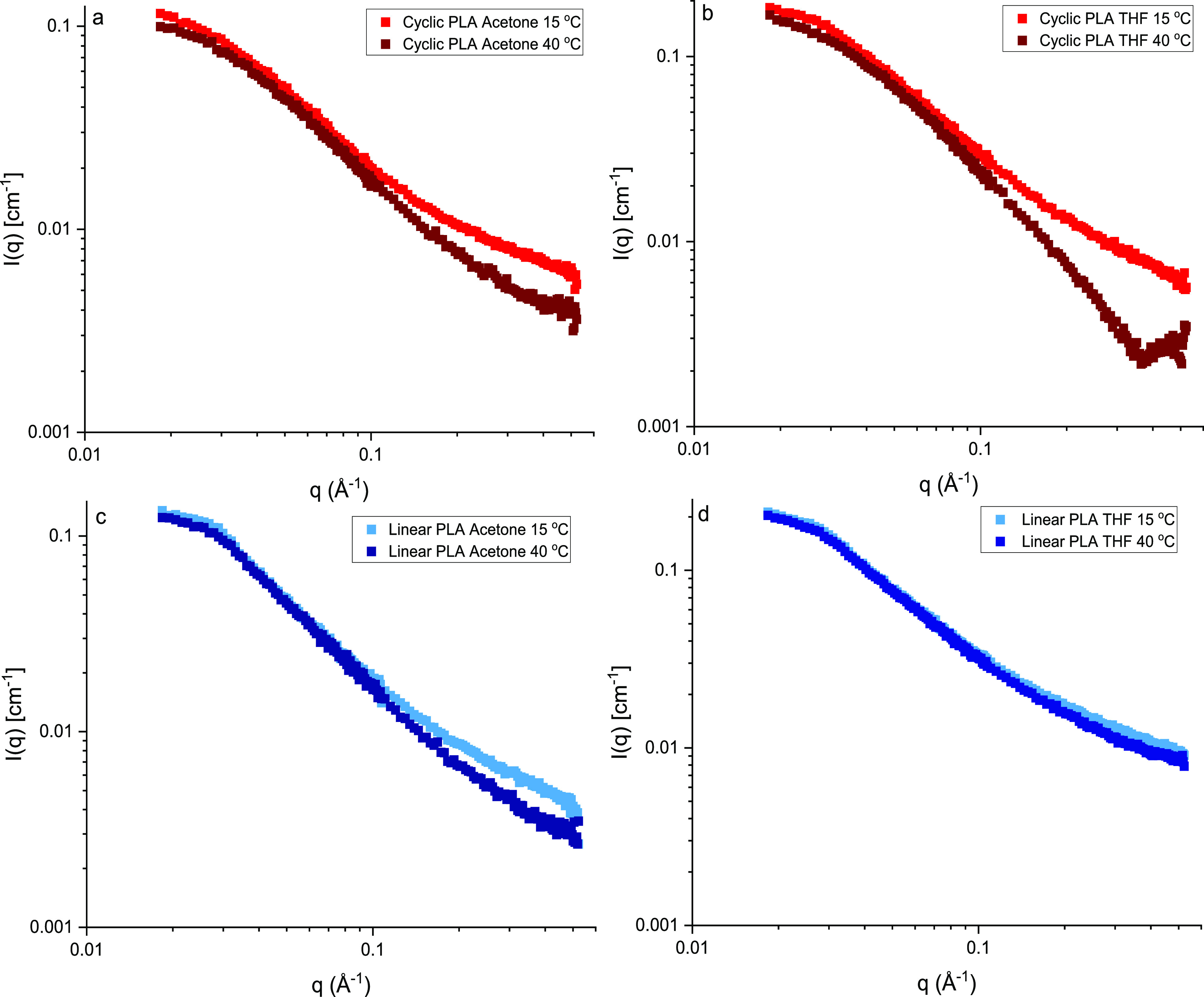
*I*(*q*) vs *q* plots of cyclic (top, red) and linear (bottom, blue) PLA samples (C1 and L1 in Table 1) in acetone-*d*_6_ and THF-*d*_8_ at 15 and 40 °C.

The *I*(*q*) profiles of key samples measured in these results can be seen in Figures 4 and 5. These show similar scattering to previous work on poly(styrene),^18^ but there are some notable observations. Full *q*-range results (Figure 4) revealed a significant upturn at low *q* for both PLA samples of similar molecular weight, implying the presence of aggregates or bubbles in solution. Low *q* measurements have been reported on cyclic poly(styrene) samples before but have not been compared directly to linear counterparts of the same molecular weight to our knowledge.^22^ For samples with a low *q* upturn, the *q* range < 0.018 Å^–1^ was excluded from fitting.

The temperature comparisons of cyclic and linear PLA samples (see Figure 5) showed that the cyclic sample was noticeably more affected by a change in temperature compared to the linear counterpart. This effect was especially distinct in the good solvent (THF-*d*_8_) and suggested that there were interactions between monomers at higher temperatures in cyclic PLA only, potentially influenced by the smaller radius of gyration of the cyclic topology. This could also be evidence of the polymer collapsing in solution sufficiently that it appears as a particle to the SANS instrument. The glass transition temperature of these samples ranged between 47 and 57 °C (see Table S1), but proximity to this temperature would not be expected to affect the behavior of the polymers in solution. The previous study by Gartner et al. reported variation in the scattering data for polystyrene at different temperatures but to a much lesser extent.^18^ This was rationalized by the formation of bubbles in the cell at temperatures close to the boiling point of the solvent. This has likely influenced the low *q* results in Figures 4 and 5, but the more substantial differences at high *q*, seen for the cyclic samples only, are not fully understood. When the data for the high-temperature sample in Figure 5b was fitted, the *q* range was restricted to exclude the upturn at high *q* (i.e., *q* > 0.3 Å^–1^ was excluded), and these data gave no anomalous fitted values.

**Figure 6:**
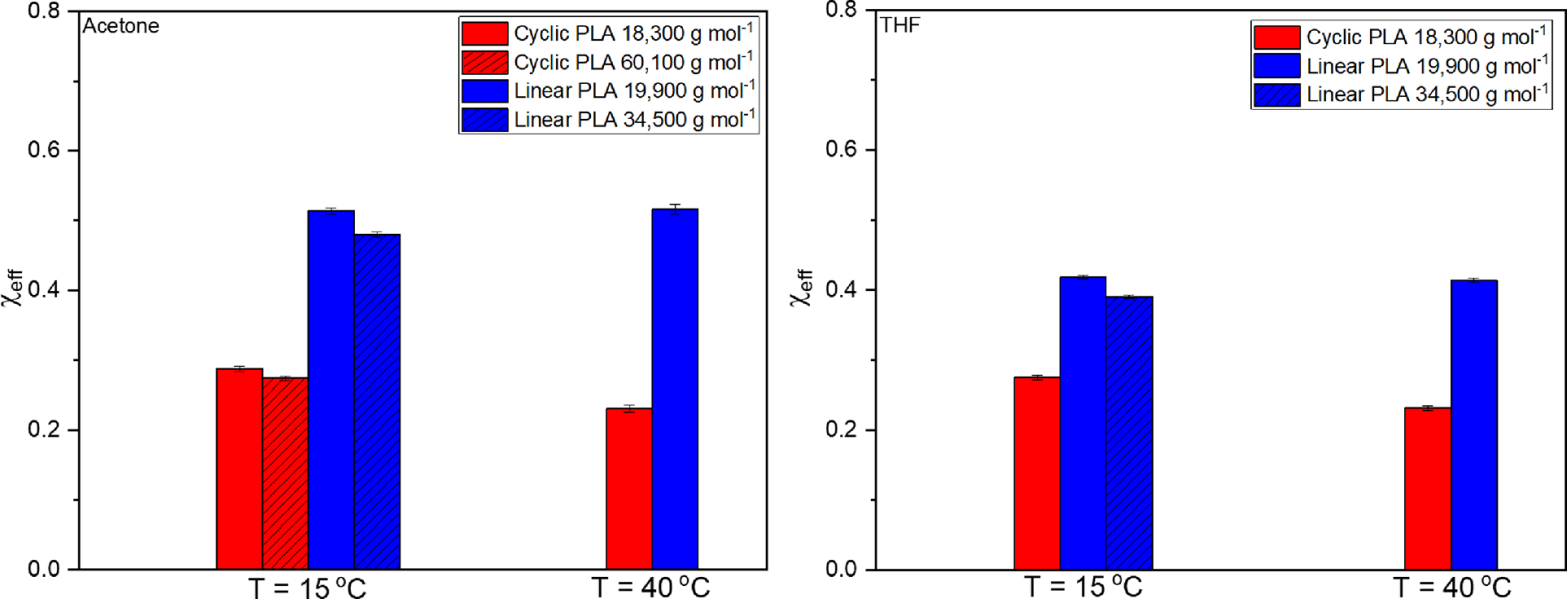
Graphs of χ_eff_ of PLA samples measured in this study at 15 and 40 °C in acetone-*d*_6_ and THF-*d*_8_. Textured bars denote different samples of the same topology as described in the legend. These data can also be seen as a graph of χ_eff_ vs 1/*T* in the Supporting Information (Figure S7).

Fitted values of χ_eff_ and ν for poly(lactide) samples showed expected trends (i.e., lower χ_eff_ and higher ν for the cyclic polymer samples), but there was a much greater difference in χ_eff_ values between the two topologies (see Figure 6). The average χ_eff_ value for the cyclic PLA samples varied between 0.23 and 0.35, whereas the range was 0.40–0.51 for corresponding linear polymers. While it was expected that the cycles would have lower χ_eff_ values, these results show differences far greater than previously reported.^18^ The ratio ⟨*R*_g, cyclic_^2^⟩/⟨*R*_g, linear_^2^⟩ for these samples was 0.841 ± 0.010, which illustrated a smaller radius of gyration for the cyclic topology as expected. Due to the easier packing and capability of hydrogen bonding, cyclic PLA may possess a higher local monomer density, and the intermolecular interactions between monomers would likely be more substantial in PLA than in polystyrene. PLA may be able to make better use of the smaller radius of gyration of the cyclic topology and further improve the solvent environment compared to linear counterparts. While there are sigma–pi interactions between chains in polystyrene, the magnitudes of the interactions between the two polymers are very different. While polymers with molecular weights ranging from 18,330 to 74,441 g mol^–1^ were analyzed, there was a minimal difference in the scattering profiles and fitted values of these polymers (as can be seen in Figure 6). This is in agreement with other SANS results, which have reported modest dependence of χ_eff_ on the molecular weight.^41^

**Figure 7:**
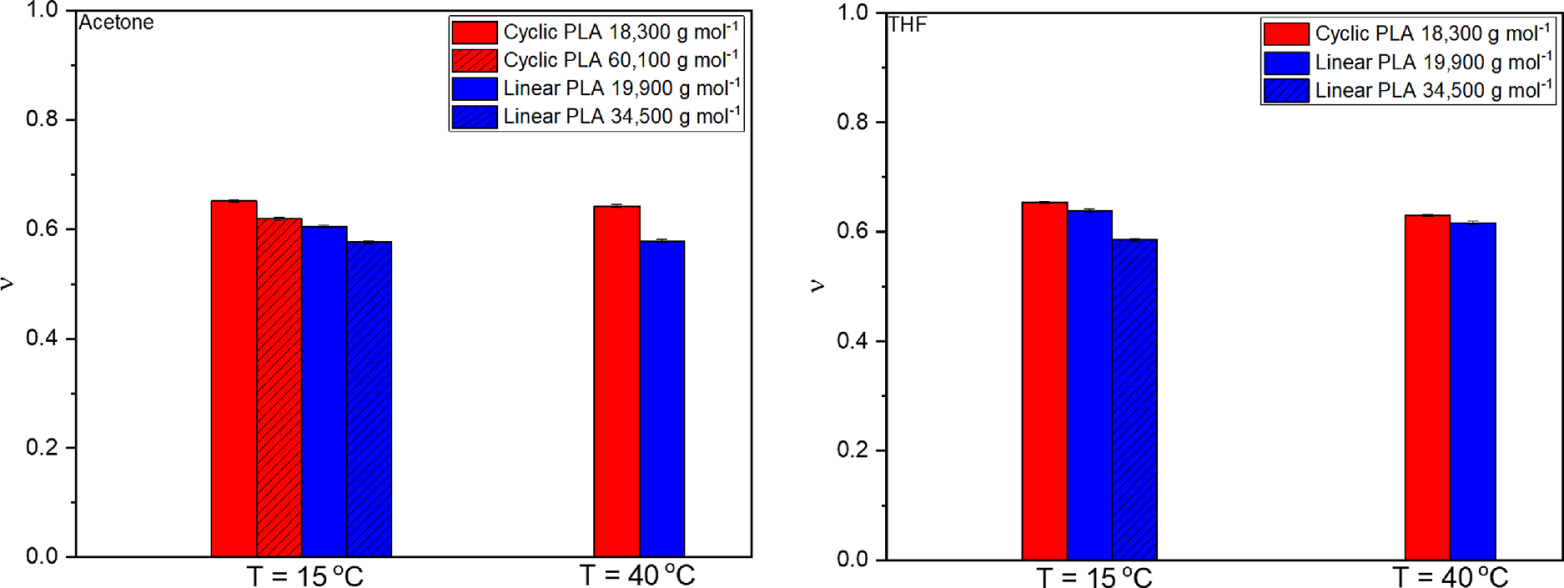
Graphs of ν against 1/*T* (Kelvin) for cyclic and linear PLA (left) samples. Textured bars denote different samples of the same topology as described in the legend. These data can also be seen as a graph of ν vs 1/*T* in the Supporting Information (Figure S8).

There was more variation in ν values for these polymers compared to the literature,^18^ although not to the same extent as for the χ_eff_ values (see Figure 7). The expected trend of a higher Flory exponent for the cyclic polymer was maintained here, while linear samples of poly(lactic acid) were more affected by solvent choice compared to cyclic counterparts.

**Figure 8:**
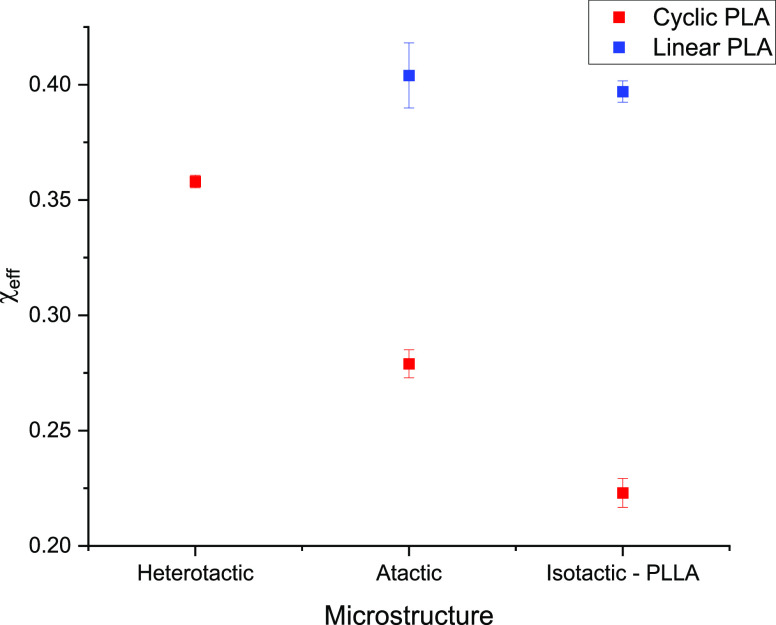
Charts showing variation in average χ_eff_ of cyclic (red) and linear (blue) PLA samples of different microstructures illustrated previously in Figure 2.

Figure 8 shows a comparison of χ_eff_ values between cyclic and linear PLA samples of differing microstructures, acquired by averaging fitted χ_eff_ values for atactic PLA samples (seen already in Figure 6), heterotactic PLA samples, and isotactic PLLA samples. This average includes polymers of varying molecular weights because this was shown to have a minimal impact on χ_eff_ values in both our and previously reported results,^41^ including all polymers allowed for more data to compare. While a minimal difference was seen in the linear polymers, there was substantial variation in cyclic PLA values, with χ_eff_ decreasing as the microstructure changed from heterotactic to atactic to isotactic PLLA (see Figure 8).

This variation in χ_eff_ across polymers of the same monomer is substantial but is not surprising given how the microstructure influences properties in PLA. A possible explanation for this may be related to the ability of the polymer chains in the different samples to pack together in the solid form. Isotactic PLLA is crystalline, with methyl groups facing the same direction allowing for close packing in a tight crystalline structure. This is well-evidenced by reported crystal structures of PLLA and the known amorphous nature of atactic and heterotactic PLA forms.^42,43^ While there are differences between packing in a crystalline phase and in solution, the behavior in solution would be expected to be influenced by differences in the crystalline phase across different forms of PLA. Any difference in the interactions between polymer chains in solution would result in a difference in local monomer density and χ_eff_ values as a result.

In contrast, heterotactic PLA is amorphous as a solid and would therefore likely also arrange less efficiently in solution, leading to fewer intrachain contacts, which would raise χ_eff_. The notable effect of the microstructure on the polymer–solvent interactions in the cyclic samples specifically can be rationalized by the smaller *R*_g_ of the cyclic topology. The increased local monomer density and intrachain contacts of the cyclic topology would lower the χ_eff_ more compared to linear counterparts but also result in a greater influence of the polymer microstructure on these results (i.e., as there are more interactions to influence). The positions of these methyl groups may also influence threading, folding, and knotting of cyclic polymers to a greater extent than in linear counterparts.

To our knowledge, there are limited reports of comparisons of χ_eff_ for linear PLA samples of differing microstructures, but it is known that amorphous PLDA (i.e., atactic or heterotactic) will exhibit a notably higher χ_eff_ value compared to crystalline isotactic PLLA (although this was not derived by SANS).^44^ In our case, we see this trend followed for the cyclic samples only, but it is still rational that the amorphous PLA would show a higher χ_eff_. Asymmetries in chain conformations among chemically similar copolymers can be known to result in relatively high χ_eff_ values due to the introduction of enthalpic and entropic contributions.^45^ The asymmetry in the atactic and heterotactic PLA samples could have contributed to higher χ_eff_ parameters.

Error in χ_eff_ and ν values was tested by setting the values of χ_eff_ and ν to different starting values: 0.25, 0.5, and 0.75 before fitting the data using the custom RPA models, which resulted in zero variation in the final fitted values. As a result, the error bars in Figures 6 and 7 were plotted using the error in the calculation reported by SasView. In the case of Figure 8, the standard deviation of χ_eff_ values was used to obtain error bars in all cases aside from isotactic PLLA samples (due to only one sample being measured, the error for this was taken from the error in the fitting of the data).

## Conclusions

We have demonstrated how SANS experiments on cyclic and linear polymers can be drastically influenced by polymer choice, microstructure, temperature, and solvent choice. The previously established trends in polymer–solvent interactions and the Flory exponent between the two topologies have been observed in our results. However, the extent of the difference in χ_eff_ between cyclic and linear poly(lactic acid) (PLA) samples was much more substantial compared to the literature on polystyrene, which illustrated how intermolecular forces and steric bulk could play a large role in the polymer–solvent interactions in cyclic polymers specifically.

Scattering profiles of *I*(*q*) vs *q* were comparable to the literature, but significant temperature variation at high *q* was observed in PLA. The study of atactic, heterotactic, and isotactic PLLA revealed substantial differences in χ_eff_ values, highlighting the extent to which cyclic PLA in particular was affected by monomer choice and microstructures, even though lactide monomers vary only in the orientation of the methyl groups.

These results highlight the value of SANS experiments in comparing cyclic and linear polymers, as well as the need to compare novel polymers such as PLA to access new variables that have not been tested before to our knowledge. Such analysis may give a better idea of the conformations and physical behavior of cyclic polymers, as well as how best to use these properties to develop cyclic polymers for specific applications as they garner increasing interest. In addition, these results highlight the potential of neutron scattering as a tool for identification and characterization of the cyclic topology.

## Supporting Information Available

Information about materials and methods used to synthesize the polymers analyzed in this work, as well as characterization data for the cyclic and linear polymers (such as MALDI-TOF spectra); further data from SANS experiments, such as some intensity vs *q* plots not included in this manuscript and *R*_g_ values for key samples (PDF)
