# Supplementary material for: Comparison of Cyclic and Linear Poly(lactide)s Using Small-Angle Neutron Scattering
Source: Macromolecules. 2022 Dec 13;55(24):11051–8. doi: 10.1021/acs.macromol.2c02020 (PMC9798859; doi:10.1021/acs.macromol.2c02020)
Supplement: Supplementary file 1 — ma2c02020_si_001.pdf [file ma2c02020_si_001.pdf]

Electronic Supplementary Information (ESI)

Comparison of Cyclic and Linear Poly(lactide)s Using Small Angle Neutron Scattering

Philip B. Yang[1]*, Matthew G. Davidson[1], Karen J. Edler[1,2]*, Niamh Leaman[1], Elly K. Bathke[1], Strachan N. McCormick[1], Olga Matsarskaia[3] and Steven Brown[4]

[1]Department of Chemistry, University of Bath, Claverton Down, Bath, BA2 7AY

[2]Lund University, SE-221 00, Lund, Sweden (current address)

[3]Institut Laue Langevin, 71 Av. Des Martyrs, 38000 Grenoble, France

[4]Scott Bader, Wollaston, Wellingborough, NN29 7RJ

Email: py300@bath.ac.uk / karen.edler@chem.lu.se

General methods and materials

All polymerizations were performed using Schlenk techniques under argon atmosphere. Racemic lactide and L-lactide were purchased from Merck and recrystallized from dry toluene, chlorobenzene was purchased from Fisher Scientific in a dry form with an Acroseal bottle. $Sn(Oct)_2$ was purchased from Merck and dried using activated molecular sieves prior to use. Benzyl alcohol, 3-methyl catechol and 4-methyl catechol were purchased from Merck and used as received. The $Sn(Oct)_2$ polymerization scheme used for atactic PLA synthesis can be seen in Figure S2. Tin-based catalysts were used to prepare cycles C1 and C2 as well as linear L1 and L2 samples. Isotactic and heterotactic samples (Cycles C3 and C4, and Linear L3) were prepared using catalysts and protocols that will be described in detail elsewhere (manuscript in preparation).

MALDI-TOF spectra were acquired using a Bruker AutoFlex Speed TOF. Samples were prepared for MALDI-TOF via the following method using THF as a solvent: 5 μL of sodium trifluoroacetate (KTFA, 2 mg mL^{-1}) was mixed with 50 μL of matrix solution (DCTB - trans-2-[3-(4-tert-butylphenyl)-2-methyl- 2-propenylidene]malononitrile, 10 mg mL^{-1}) to suppress the formation of other (potassium, lithium) ions. 20 μL of dissolved samples (5 mg mL^{-1}) were added to the matrix-salt mixture and 1 μL of the mixed solution was deposited on a stainless-steel target.[1]

An Agilent 1260 Infinity Multi-Detector GPC was used to gauge relative molecular weights reported in Table 1 (to a poly(styrene) standard) using refractive index methods. For Mark-Houwink plots (i.e. Figure 4), a combination of refractive index, light scattering and viscosity measurements were used to generate the plot of intrinsic viscosity versus absolute molecular weight. Two columns are used to separate polymer chains by molecular weight: a PLgel 5 µm MIXED-D 300 x 7.5 mm, with a guard column PLgel 5 µm MIXED Guard 50 x 7.5 mm. All samples were dissolved in GPC grade THF (2 mg ml^{-1}) and left overnight before being passed through a hydrophilic filter. The injected sample volume was 100 µL, the flow rate of the instrument was 1 mL min^{-1} and the measurements were conducted at 35 °C.

NMR spectra were acquired on Bruker Avance III 400 and 500 MHz spectrometers Spectra were referenced to solvent peak for $CDCl_3$.[2] To determine tacticity of PLA samples, the probability of racemic enchainment (P_r) was calculated using homonuclear decoupling experiments on the methine region of the PLA NMR spectra. Each decoupled peak in this region can be attributed to a specific arrangement of stereocenters according to literature. P_r values represent the degree of heterotactic arrangement in the polymer. A value of 1 refers to a fully heterotactic polymer.[3]

The DSC model was a TA instruments DSC Q20. DSC measurements of poly(ε-decalactone) samples involved two heating and cooling cycles between -70 and 50 °C with a heating/cooling rate of 20°C min^{-1}. Reported T_g's in Table 1 were taken from the second curve in the resulting DSC trace, after any thermal history had been cleared.

Polymer Characterization

Cyclic purity was verified by a combination of characterization techniques including: differences in viscosity as seen in Mark-Houwink plots generated from triple-detection GPC (see Figure S2); lack of end groups in both ^{1}H NMR and MALDI-TOF spectra (see Figures S3, S4, S5 and S6); and increases in glass transition temperatures of cyclic vs. linear polymers (the glass transition temperatures of the cyclic PLA samples were 4-5 °C higher than comparable linear counterparts – see Table 1 in main text), which is indicative of cyclic topology.

Tacticity was determined using homonuclear decoupling NMR experiments on peaks for the methine region of PLA (see Figure S7). The ratio of peak intensities (which correspond to the difference possible stereosequences of monomers) was used to calculate the probability of racemic enchainment (P_r), giving the degree of heterotacticity between 0 (fully isotactic), 0.5

(fully atactic) and 1 (fully heterotactic). P_r values of 0.77 for cyclic sample L4 indicated a strong heterotactic preference in the polymer. This allowed for comparison with the more atactic polymers made using $Sn(Oct)_2$/catechol system.[3–5]

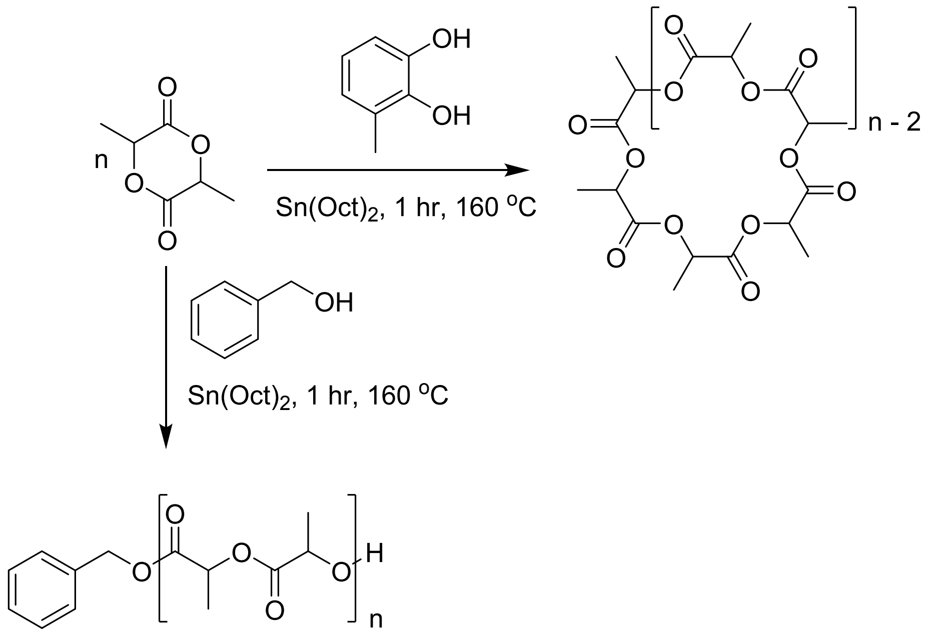

Figure S1: Reaction scheme for atactic polymerizations of lactide using $Sn(Oct)_2$ for both cyclic and linear topologies through variation of co-initiator.

Mark-Houwink Plots and MALDI-TOF spectra – PLA

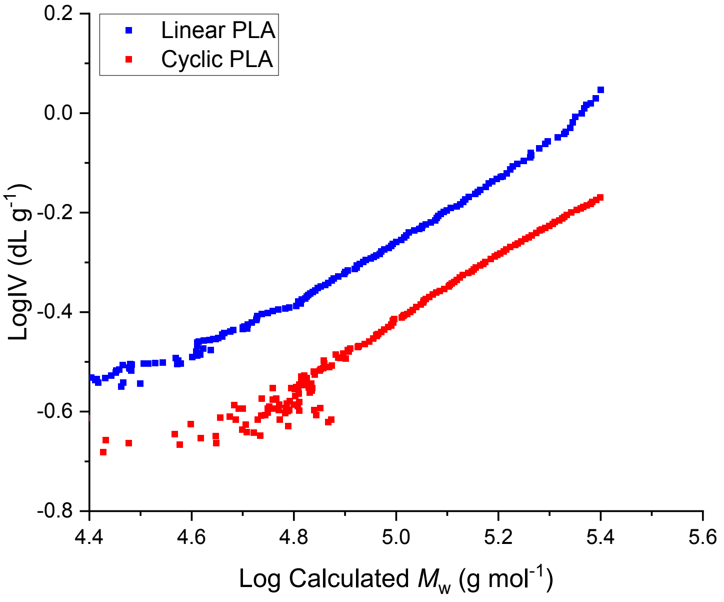

$\eta_{cyclic} / \eta_{linear}$ was estimated to be 0.70

Figure S2: Log(intrinsic viscosity) *vs* log(M_w) plot comparing cyclic and linear PLA samples.

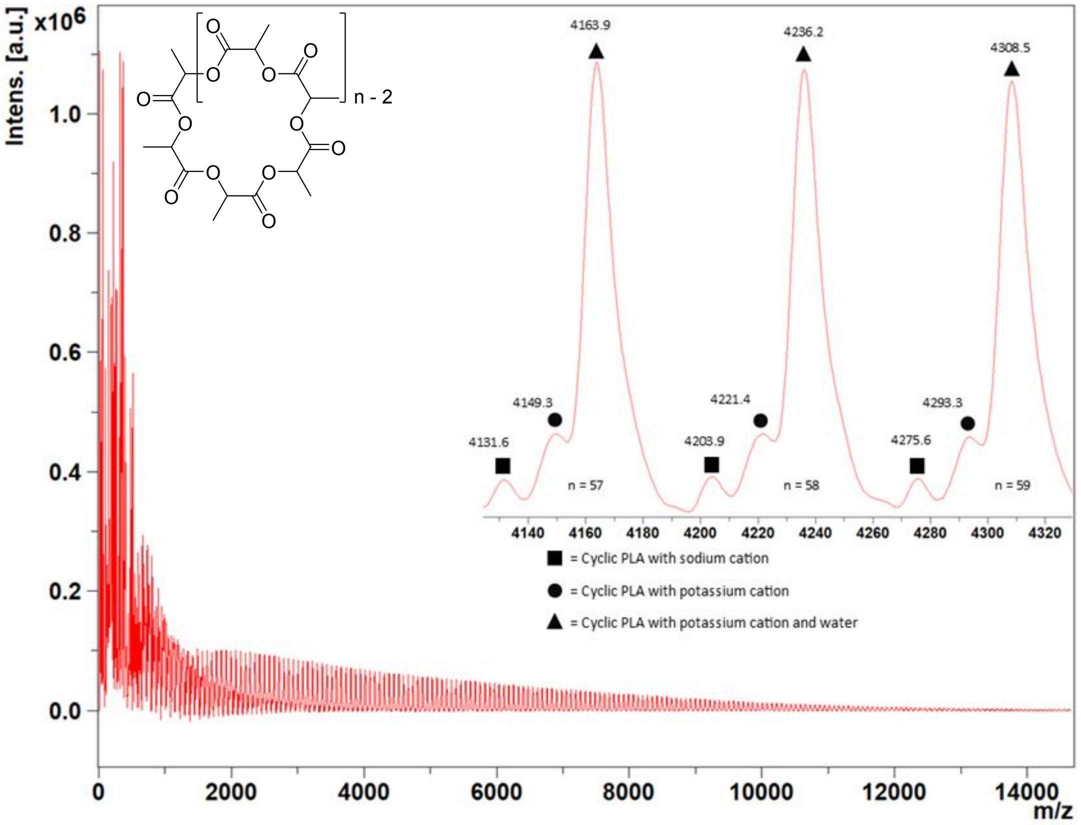

Figure S3: MALDI-TOF spectra of a cyclic PLA sample, showing peaks for cyclic species only with various different counterions.

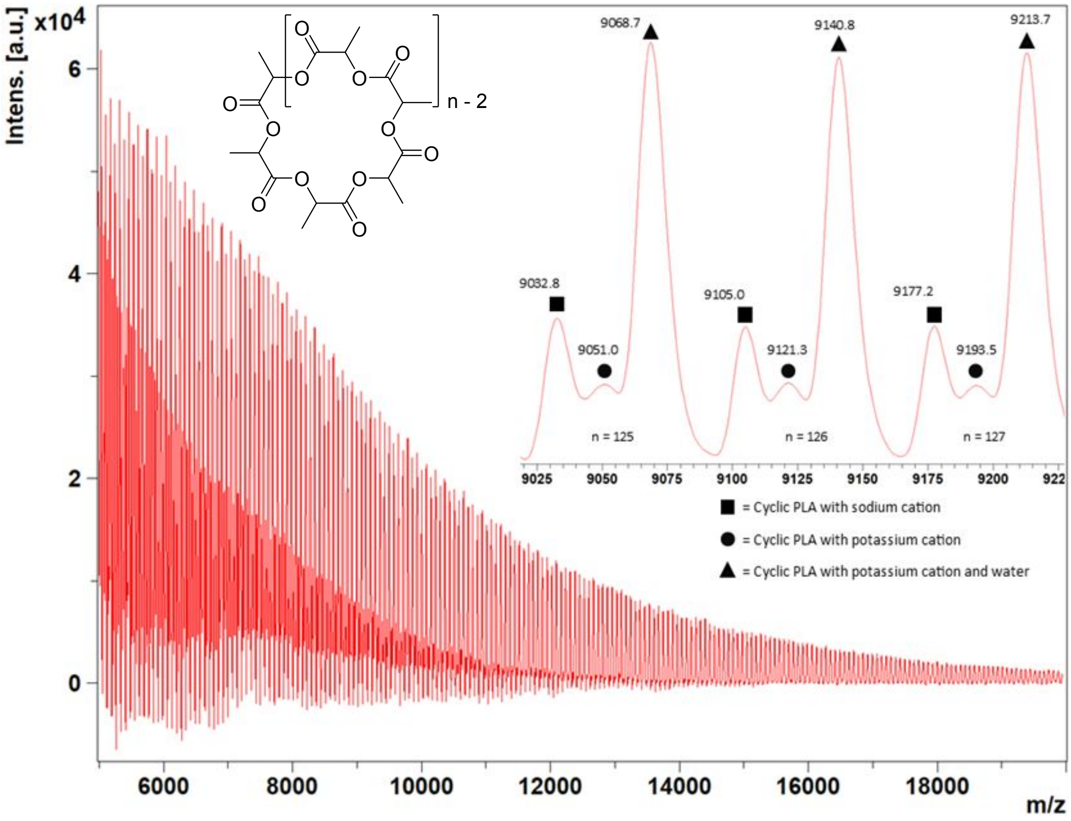

Figure S4: MALDI-TOF spectra of a high molecular weight cyclic PLA sample, showing peaks for cyclic species only with various different counterions.

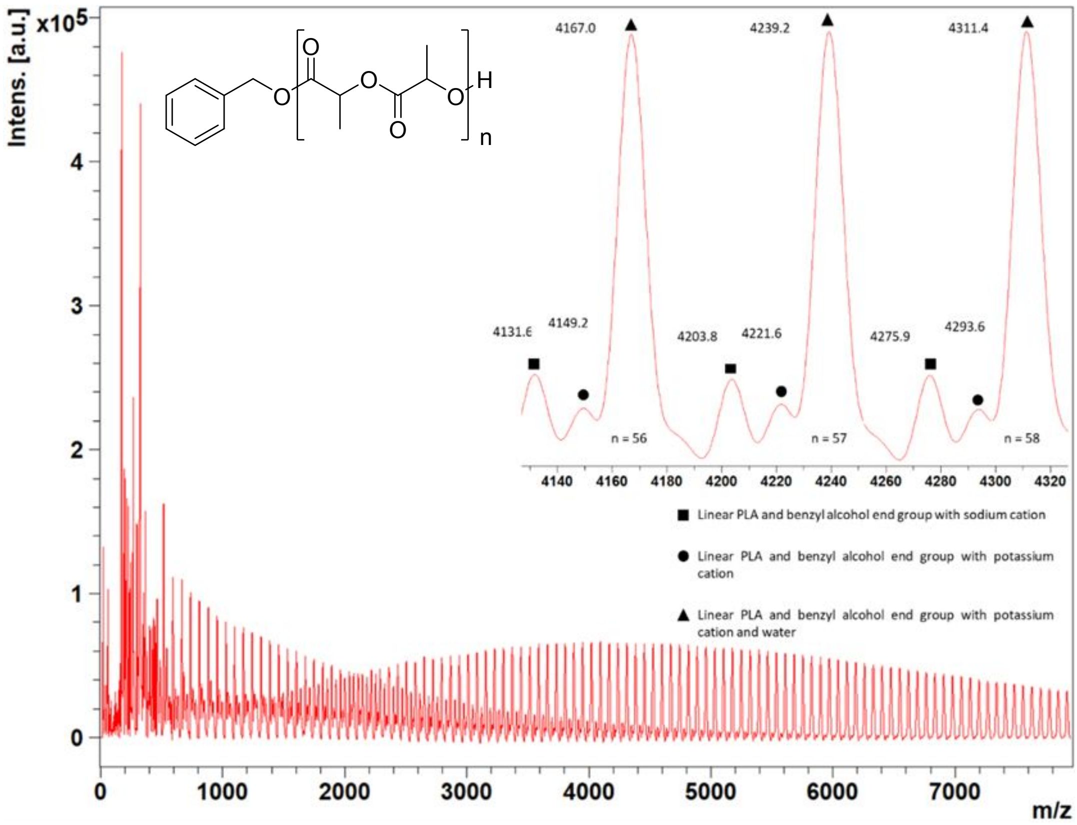

Figure S5: MALDI-TOF spectra of a linear PLA sample, showing mostly peaks for linear polymer chains initiated with benzyl alcohol, the co-initiator for this reaction – cyclic species can be seen to be dominant at low molecular weights.

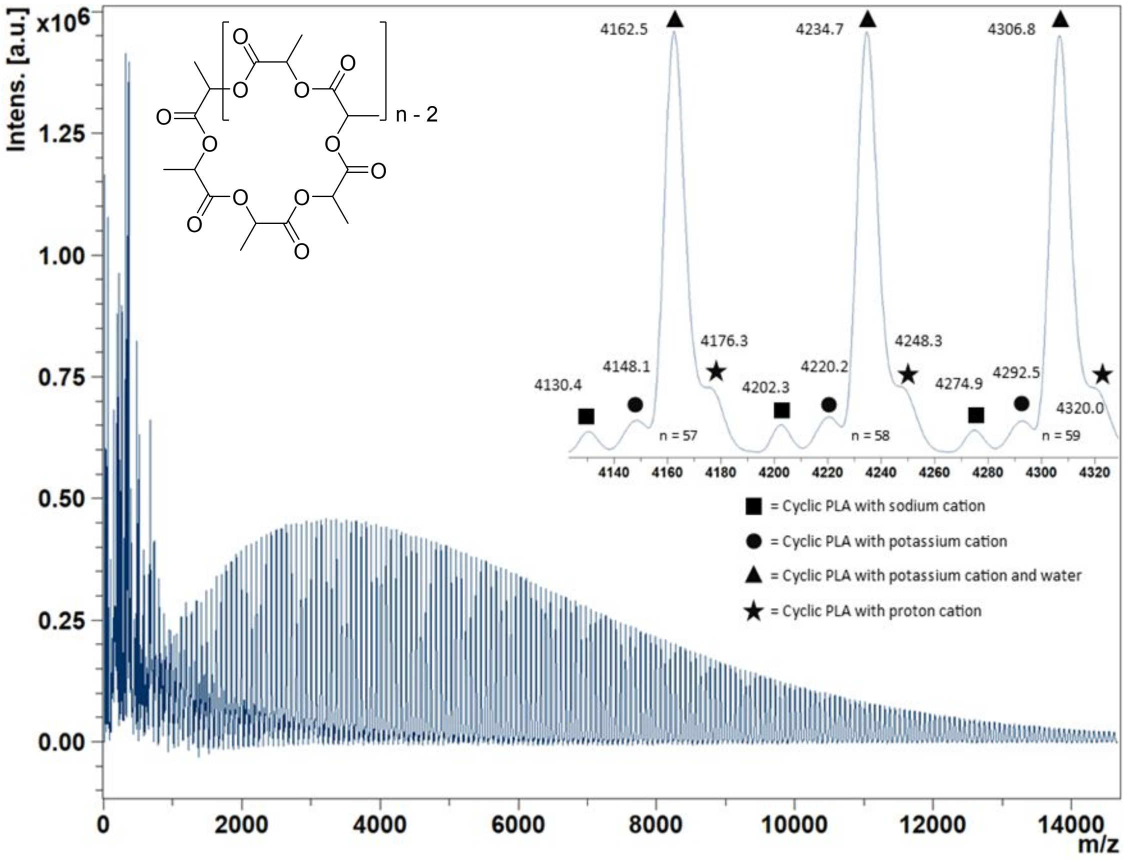

Figure S6: MALDI-TOF spectra of a cyclic PLA sample, showing peaks corresponding to cyclic species with no end groups and various different counterions. The shoulder peaks (denoted with star symbols) were not observed in other cyclic MALDI-TOF spectra but were assigned as cyclic polymer species. This was due to the well-known effects of linear contaminants on cyclic polymer viscosity, which were not observed in these samples. Cyclic purity was further confirmed in other MALDI-TOF spectra (see Figures S3 and S4) as well as viscosity and T_g differences with comparable [illegible]

Additional data from SANS (e.g. Intensity vs. q plots, fit examples, R_g data)

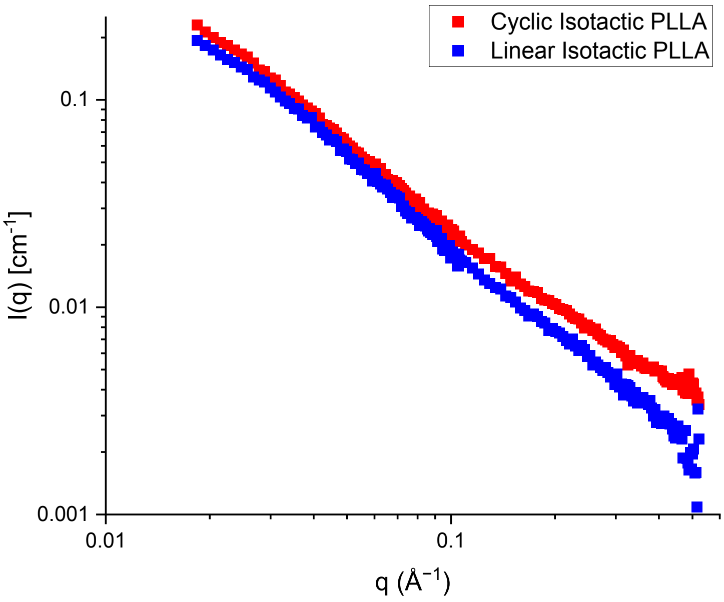

Figure S7: Intensity vs. q plot comparing Isotactic PLLA samples Cycle L3 and Linear L3 in THF-d^8 at 15 °C.

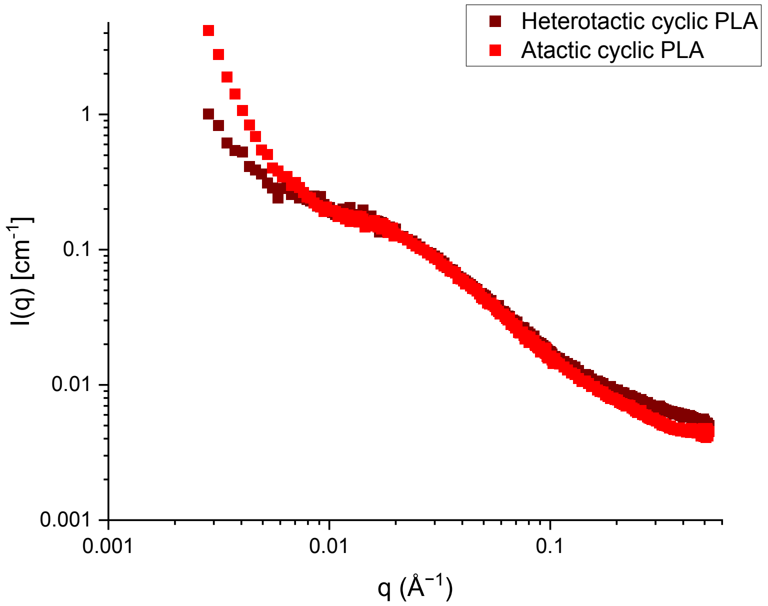

Figure S8: Intensity vs. q plot comparing heterotactic and atactic samples Cycle C2 and Cycle C4 in acetone-d^6 at 15 ºC.

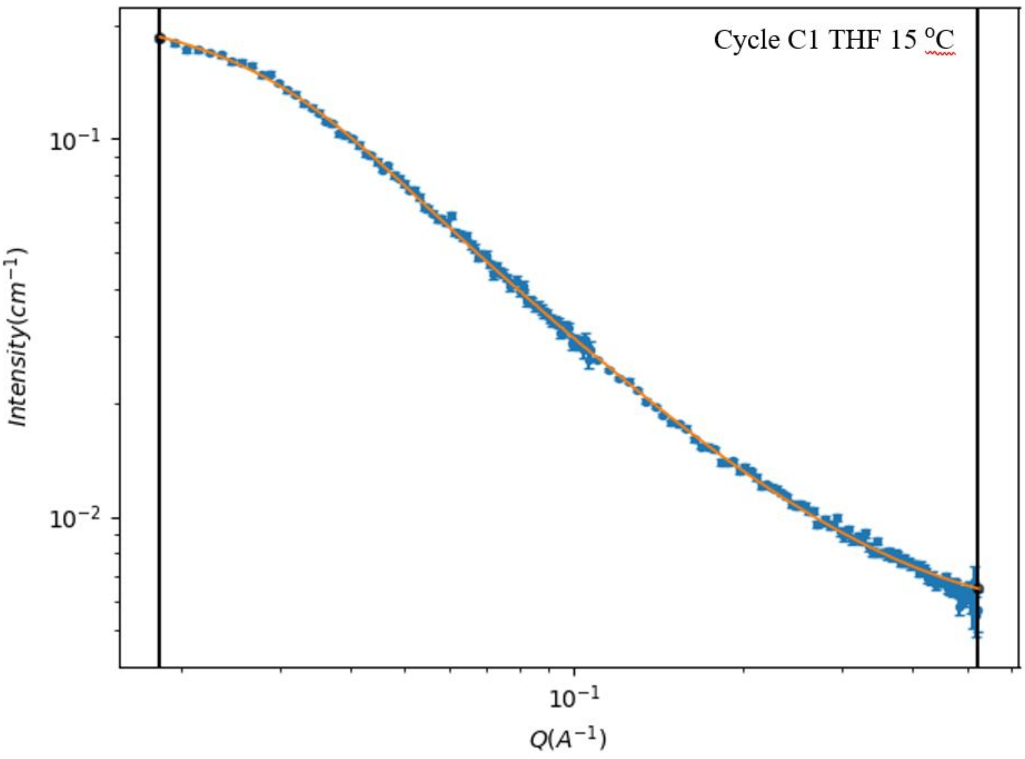

Figure S9: Intensity vs. q plot of sample Cycle C1 in THF at 15 °C with fit line (orange) from RPA model.

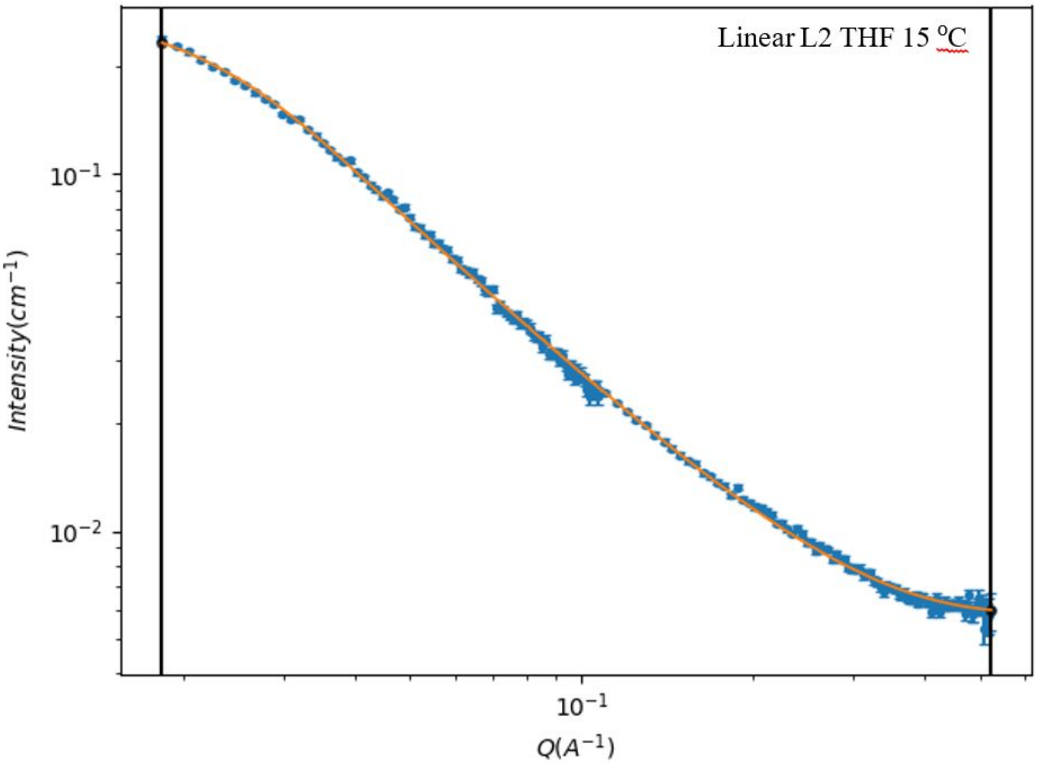

Figure S10: Intensity vs. q plot of sample Linear L2 in THF at 15 ºC with fit line (orange) from RPA model.

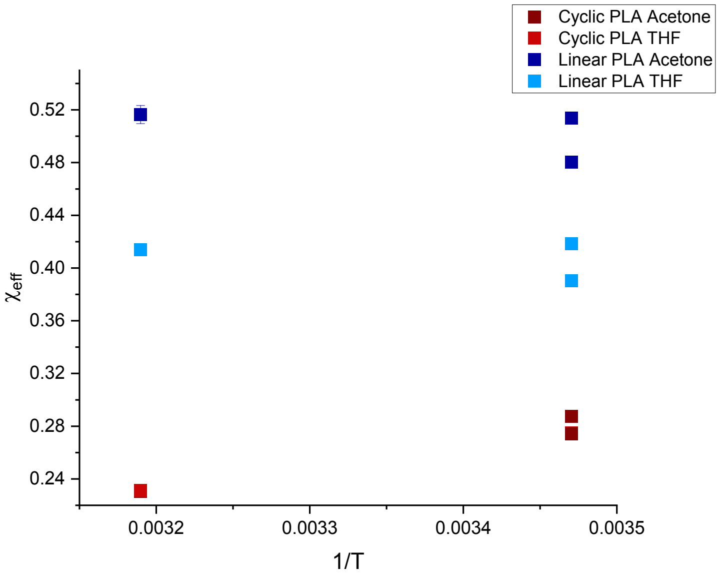

Figure S11: Graph of χ_{eff} vs. 1/T for cyclic and linear PLA samples measured in this study in both Acetone-d[6] and THF-d[8].

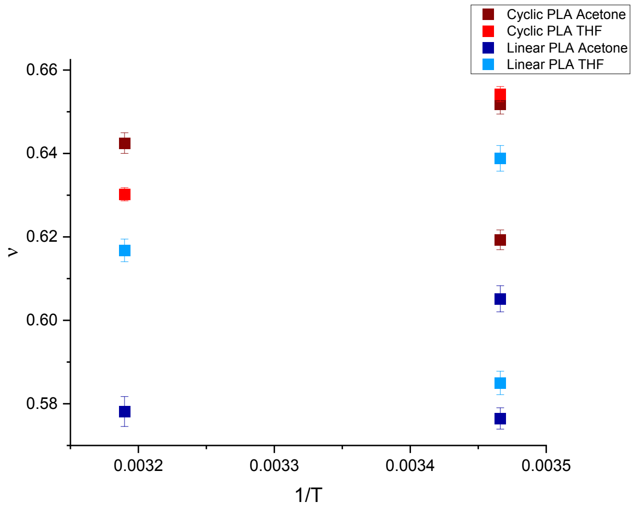

Figure S12: Graph of ν vs. 1/T for cyclic and linear PLA samples measured in this study in both Acetone-d[6] and THF-d[8].

Table S1: Comparison of fitted results of Cycle C1 in THF at 15 °C with volume fraction as a fitted/constant parameter

**Volume fraction state**	**ϕ_p**	**ϕ_p error**	**χ_{eff}**	**χ_{eff} error**	**ν**	**ν error**
**Constant**	0.0100	N/A	0.2723	0.0032	0.6617	0.0007
**Fitted**	0.0091	0.0001	0.2751	0.0031	0.6543	0.0018

Table S2: Radii of gyration for Cyclic and Linear PLA samples calculated from Guinier plots

Sample	Solvent	Temperature (°C)	R_g (Å)
C1	Acetone	15	38.3 ± 0.4
C1	Acetone	40	37.0 ± 0.5
C1	THF	15	38.7 ± 0.4
C1	THF	40	37.9 ± 0.4
C2	Acetone	15	46.3 ± 0.6
C3	THF	15	48.3 ± 0.8
C4	Acetone	15	46.4 ± 0.6
C4	THF	15	45.4 ± 0.7
L1	Acetone	15	42.8 ± 0.7
L1	Acetone	40	41.5 ± 0.5
L1	THF	15	41.7 ± 0.4
L1	THF	40	40.9 ± 0.2
L2	Acetone	15	48.3 ± 0.7
L2	THF	15	44.7 ± 0.7
L3	THF	15	46.6 ± 0.8

References

(1) Kricheldorf, H. R.; Weidner, S. M. SnOct2-Catalyzed Syntheses of Cyclic Poly(l-Lactide)s with Catechol as Low-Toxic Co-Catalyst. *J. Polym. Environ.* **2019**, *27* (12), 2697–2706. https://doi.org/10.1007/s10924-019-01545-5.

(2) Fulmer, G. R.; Miller, A. J. M.; Sherden, N. H.; Gottlieb, H. E.; Nudelman, A.; Stoltz, B. M.; Bercaw, J. E.; Goldberg, K. I. NMR Chemical Shifts of Trace Impurities: Common Laboratory Solvents, Organics, and Gases in Deuterated Solvents Relevant to the Organometallic Chemist. *Organometallics* **2010**, *29* (9), 2176–2179. https://doi.org/10.1021/om100106e.

(3) Chamberlain, B. M.; Cheng, M.; Moore, D. R.; Ovitt, T. M.; Lobkovsky, E. B.; Coates, G. W. Polymerization of Lactide with Zinc and Magnesium β-Diiminate Complexes: Stereocontrol and Mechanism. *J. Am. Chem. Soc.* **2001**, *123* (14), 3229–3238. https://doi.org/10.1021/ja003851f.

(4) Mann, J. W. A. and B. E. NMR and Chemistry: An Introduction to Modern NMR Spectroscopy, 4th Ed. **2000**, 400.

(5) Zell, M. T.; Padden, B. E.; Paterick, A. J.; Thakur, K. A. M.; Kean, R. T.; Hillmyer, M. A.; Munson, E. J. Unambiguous Determination of the 13C and 1H NMR Stereosequence Assignments of Polylactide Using High-Resolution Solution NMR Spectroscopy. *Macromolecules* **2002**, *35* (20), 7700–7707. https://doi.org/10.1021/ma0204148.
